# Characteristics and Outcomes Associated With Emergent Rectal Foreign Body Management: A Retrospective Cohort Analysis

**DOI:** 10.7759/cureus.49413

**Published:** 2023-11-25

**Authors:** Eric Frendt, Momin Masroor, Arman Saied, Arianna Neeki, Santana Youssoffi, Aldin Malkoc, Fanglong Dong, Louis Tran, Rodney Borger, David T Wong, Michael Neeki

**Affiliations:** 1 Emergency Department, Arrowhead Regional Medical Center, Colton, USA; 2 Emergency Department, California University of Science and Medicine, Colton, USA; 3 Surgery, Arrowhead Regional Medical Center, Colton, USA

**Keywords:** duration of hospital stay, colonic perforation, rectal perforation, colonic foreign body, rectal foreign body

## Abstract

Background

Bedside management and outcomes of rectal foreign bodies remain challenging due to the presentation and complexity of the inserted objects. Injuries, such as perforation of the colon and rectum, are among the most commonly reported complications. However, prior studies are unclear regarding the setting in which the complication rates may be minimized. This study aimed to assess whether there was a statistically significant difference among the various extraction methods with regard to complications in the emergency department and operating room.

Materials and methods

This was a retrospective study of all cases of rectal foreign bodies that were removed in the emergency department at a large county hospital between 1/1/2010 and 12/31/2020. Patients included in this study were adults who were evaluated and treated in the emergency department.

Results

A total of 78 patients were included in the final analysis. More than half (51.3%, n=40) of the patients were successfully treated in the emergency department. Compared with the emergency department, patients in the operating room were more likely to undergo exploratory laparotomy and colectomy (0% vs. 31.6%, p<0.0001), undergo general anesthesia (84.2% vs. 0%, p<0.0001), have higher complication rates (21% vs. 0%, p=0.0021), and have a longer hospital length of stay (median=1 vs. 0, p<0.0001).

Conclusion

This study revealed a >50% success rate of rectal foreign body removal in the emergency department without any reported complications. To improve the success rate of bedside retrieval and decrease complications, physicians need to be vigilant, communicative, and compassionate about their evaluations and clinical methodology.

## Introduction

The management of rectal foreign bodies (RFBs) presents unique challenges in the emergency department (ED). RFBs may consist of various compositional materials and ergonomics, including but not limited to vegetables, light bulbs, everyday household items, and body packing of illicit drugs [1-3]. Patients presenting to the ED with RFBs range widely in age from prepubertal to older adults [4,5]. The insertion of RFBs has been classified as voluntary or involuntary, as well as sexual or non-sexual [6]. The definition of RFBs can be blurry because many objects inserted via the rectum are large enough to enter the sigmoid colon [7-9]. Although detailed epidemiologic data are scant, recent studies have reported a progressive rise in complications related to RFBs, with a disproportionately higher incidence in men and an average age in the mid-40s [6,10-12].

Lake and colleagues noted that nearly 75% of all RFBs, both rectal and colonic, could be safely removed at the bedside [13]. Furthermore, operative intervention was required in 55% of patients with RFB reaching the sigmoid colon versus 24% for patients with objects located in the rectum [13]. A similar study reported a success rate of 96% for bedside removal of the inserted objects digitally, and the rest were removed laparoscopically in the operating room (OR) [14]. Barone et al. noted an 88% success rate in digital extraction of RFBs at the bedside, while the other 12% required laparoscopic removal in the OR [15]. A few investigators have also suggested an algorithm for approaching RFB removal starting from the least invasive technique of transanal extraction using tools such as forceps for operative procedures such as exploratory laparotomy [6]. Some of the previously reported methods for the removal of RFBs include the use of surgical forceps [5,16], obstetric vacuum [17,18], and Foley catheters passing the object from outside or placed inside the object's cavity [2,19]. In addition, procedural sedation has been reported to be adequate for most cases before bedside transanal extractions [13,20].

RFB has been reported to result in a variety of complications [1,2,21]. According to the Rectal Organ Injury Scale (ROIS) proposed by the American Association for the Surgery of Trauma, injuries caused by RFBs range from grade I to grade 22. Grade I injury was defined as hematoma without devascularization. Grade II injuries include partial thickness laceration of less than 50% of the circumference, while grade III injuries demonstrate partial thickness laceration greater than 50% of the circumference. Grade IV injuries are full-thickness lacerations that extend into the perineum. Grade V injury demonstrates a de-vascularized segment [22]. Colonic organ injury scale grading is very similar to ROIS, with differences consisting of transection of the colon in grade IV and transection of the colon with a de-vascularized segment and tissue loss in grade V [22]. Previous studies suggest that most injuries resulting from inserted objects are classified as grade I injuries [22].

Investigators from two separate studies suggested a complication rate of nearly 10% in cases of attempted transanal bedside removal in the ED [4,14]. The most commonly reported complication of bedside procedures is perforation of the rectum [2,19,23], followed by rectal mucosal injury [1,16]. One study noted that 17% of the patients sustained perforation of the colon [21]. The underlying cause for such high rates of complications may be multifactorial, including the status of the patients, the presence of comorbidities, and delays in presentation to the hospital [2,4,19]. To reduce the rate of complications, Ploner et al. discussed various extraction methods, such as sigmoidoscopy [24].

With the increased incidence of RFB and its associated complications, it is imperative to explore and determine standards for the safe and effective removal of RFBs. However, the current literature is not clear about the ideal setting. This study aimed to further explore various extraction methods, outcomes, and complications associated with bedside attempts at the removal of RFBs.

## Materials and methods

This was a retrospective study of all cases of RFBs that were removed in the ED at a large county hospital from 1/1/2010 to 12/31/20. The patients included in this study were adults aged ≥18 years who were evaluated and treated in the ED. Patients diagnosed with RFB as the primary diagnosis were identified using the International Classification of Disease, Ninth and Tenth Revision (ICD-9, ICD-10) codes [25]. Patient demographics in this study were extracted from their electronic medical records and included race, marital status, and insurance status. Informed consent was waived, and data were reported in an aggregated format. No patient data were included in this study. Consent, ethical approval, data gathering, and analysis were obtained from the local institutional Institutional Review Board (IRB #20-46). This study was reported in accordance with the Strengthening The Reporting Of Cohort Studies in Surgery (STROCSS) 35 criteria. This project has been registered with ClinicalTrials.org. 

Patients were identified as having their RFB successfully removed in either the ED or the operation room (OR). Patients involved had few comorbidities, and all patients were physically fit for surgery if required. If the initial removal attempt in the ED was unsuccessful, an attempt was made by the surgery team in the OR. Two groups were created for statistical analysis: those who had the RFB successfully removed in the ED and those who were attempted in the ED but were later transferred to the OR. The two extraction methods identified in the ED setting were digitally/forceps or a Foley catheter. Additionally, the OR setting included exploratory laparotomy/colectomy and sigmoidoscopy as possible extraction methods. Neither group included patients who presented with acute abdomen or intra-abdominal sepsis. Our exclusion criteria were all patients who were prisoners, cognitively impaired, pregnant females, and anyone under the age of 18.

All statistical analyses were conducted using the SAS software for Windows version 9.3 (SAS Institute Inc., Cary, North Carolina). Descriptive statistics are presented as means and standard deviations for continuous variables, along with frequencies and proportions for categorical variables. An intense T-test was conducted to assess the difference in continuous outcomes between the ED and OR. Wilcoxon rank-sum tests were conducted to assess the difference in the median of non-normal continuous variables. Chi-squared tests were used to assess the association between categorical variables and location (ED vs. OR). Fisher’s exact test was used if the expected cell count for each cell was less than 5. All statistical tests were performed on both sides. Statistical significance was set at p<0.05.

## Results

A total of 78 patients with a confirmed diagnosis of RFB were included in the final analysis. The average age was 43.4 (SD=13.2) years. The majority of these patients were male (n=69, 88.5%), with half (50%, n=39) of the patients being Hispanics. Almost three-quarters (74.6%, n=58) of the participants had Medi-Cal insurance. Table 1 presents a detailed demographic summary of patients' characteristics.

**Table 1:** Demographic summary of the 78 patients with a confirmed diagnosis of rectal foreign body ED - patients were treated in the ED with successful rectal foreign body removal; OR - patients were initially treated in the ED but were later transferred to the OR for the rectal foreign body removal

Characteristics	N	Proportion
Gender		
Female	9	11.50%
Male	69	88.50%
Ethnicity		
Black	8	10.30%
Hispanic	39	50.00%
White	31	39.70%
Insurance		
Medi-Cal	58	74.40%
Private insurance	9	11.50%
Uninsured	11	14.10%
Location of successful extraction		
ED	40	51.30%
OR	38	48.70%
Methods of removal		
Digital and forceps/anoscopy	53	68.00%
Exploratory laparotomy/colectomy	12	15.40%
Foley/forceps	3	3.90%
Sigmoidoscopy	10	12.80%
Complications		
Air leakage along the suture line	1	1.30%
Anal fissure	1	1.30%
Internal sphincter tear	1	1.30%
Rectal mucosal injury	1	1.30%
Bowel perforation	3	3.90%
Postoperative ileus	1	1.30%
No complication	70	89.70%
Type of anesthesia		
Procedural sedation	46	59.00%
General anesthesia	32	41.00%
Age	43.4 ± 13.2	
Hospital length of stay	1 (0, 1)	

Among 78 patients who required RFB removal, more than half (51.3%, n=40) were treated in the ED with successful transanal extraction. The remaining patients (48.7%, n=38) were subsequently transferred to the OR for further intervention. The most common removal methods were digital and forceps/anoscopy (n=53, 68.0%). The median length of hospital stay was one day, with the first and third quartiles being zero and one day, respectively. Most (89.7%, n=70) of the patients had no reported complications. Eight complications were reported, including air leakage along the suture line (n=1), anal fissure (n=1), internal sphincter tear (n=1), rectal mucosal injury (n=1), bowel perforation (n=3), and postoperative ileus (n=1). Moreover, more than half (59.0%, n=46) of the patients received procedural sedation while undergoing bedside procedures.

Table 2 compares the characteristics of patients who were successfully treated in the ED and those who required OR intervention. There were no statistically significant differences in the demographic variables between ED and OR, such as sex (p=0.155), ethnicity (p=0.1287), and insurance type (p=0.335). However, there was a statistically significant difference between the two groups in terms of age (p=0.0439) and the methods of removal of RFB (p<0.0001). Compared with patients in the ED, those in the OR were more likely to undergo exploratory laparotomy and colectomy (0% vs. 31.6%, p<0.0001). Moreover, patients transferred to the OR were also more likely to undergo general anesthesia (84.2% vs. 0%, p<0.0001), had higher complication rates (21% vs. 0%, p=0.0021), and had a longer hospital length of stay (median=1 vs. 0, p<0.0001).

**Table 2:** Comparison of demographics and outcomes between the two cohorts ED - patients were treated in the ED with successful rectal foreign body removal; OR - patients were initially treated in the ED but were later transferred to the OR for the rectal foreign body removal P-value was calculated by creating binary variables indicating whether there was a complication using Fisher's exact test

Characteristics	ED (n=40)	OR (n=38)	p-value
Gender			0.155
Female	7 (17.5%)	2 (5.3%)	
Male	33 (82.5%)	36 (94.7%)	
Ethnicity			0.1287
Black	6 (15%)	2 (5.3%)	
Hispanic	22 (55%)	17 (44.7%)	
White	12 (30%)	19 (50%)	
Insurance			0.335
Medi-Cal	28 (70%)	30 (79%)	
Private insurance	4 (10%)	5 (13.2%)	
Uninsured	8 (20%)	3 (7.9%)	
Methods of removal			< .0001>
Digital and forceps/anoscopy	38 (95%)	15 (39.5%)	
Exploratory laparotomy/colectomy	0 (0%)	12 (31.6%)	
Foley/forceps	2 (5%)	1 (2.6%)	
Sigmoidoscopy	0 (0%)	10 (26.3%)	
Complications			0.0021
Air leakage along the suture line	0 (0%)	1 (2.6%)	
Anal fissure	0 (0%)	1 (2.6%)	
Internal sphincter tear	0 (0%)	1 (2.6%)	
Rectal mucosal injury	0 (0%)	1 (2.6%)	
Bowel perforation	0 (0%)	3 (7.9%)	
Post-operative ileus	0 (0%)	1 (2.6%)	
No complication	40 (100%)	30 (79%)	
Type of anesthesia			< .0001>
General anesthesia	0 (0%)	32 (84.2%)	
Procedural sedation	40 (100%)	6 (15.8%)	
Age	40.5 ± 12.8	46.5 ± 13.2	0.0439
Hospital length of stay	0 (0, 0.5)	1 (1, 4)	< .0001>

## Discussion

The emergent presentation of RFB cases can be very elusive based on the patient's condition and willingness to share a detailed history. The majority of RFBs are reported to be self-inserted [1,3]; however, it is often very embarrassing for patients to openly admit them during the initial encounter in a hospital setting. One study reported that approximately 20% of patients presented to the ED without an initial complaint of RFB [12]. As a result, physicians need to be more receptive and vigilant when investigating possible RFB insertion based on history and physical examination. Common physical signs and symptoms associated with this condition include rectal pain, constipation, bright red blood per rectum, fecal incontinence, and peritoneal signs [6]. It is imperative to plan a safe strategy before removing RFBs in anticipation of possible progressive injuries. As such, our primary study focused on determining the safest option for the removal of rectal foreign bodies.

It is important for physicians to consider cases of suspected sexual abuse [11]. Involuntary insertion of RFBs has been reported in children, the elderly, and in individuals with mental disabilities. In cases of sexual abuse, special attention should be paid to addressing both the mental and physical well-being of the patient [19]. Many victims of sexual abuse may be unwilling to undergo examinations. Therefore, it is important for physicians to consider following a trauma-informed care approach when interviewing and examining potential sexual assault victims [26]. Some of the elements included asking the patient to disrobe only when necessary or to disrobe only partially, obtaining consent for each part of the examination performed, and asking the patient if there was any way to make the procedure easier for them [26]. Special concern must also be taken to avoid contamination of potential evidence [27]. The suggested guidelines will help the ED care team provide appropriate care for sexual assault victims with RFBs and ease their suffering [26]. Additionally of note, there have been thoughts that patients without insurance do not seek medical attention. In our study, we noted that 14% of patients did not have insurance.

This study revealed a 51.3% success rate of RFB removal in the ED without any reported complications. The remaining patients underwent further emergent re-evaluation in the setting of OR utilizing general anesthesia along with exploratory laparotomy, colectomy, and/or sigmoidoscopy based on the location and shape of the objects. The success rate in this study was lower than that reported in a previous study, which reported a 74% bedside extraction success rate without complications [13]. This discrepancy may be attributed to the fact that the majority of the objects encountered in the current study may have been located in the colon. As noted by Lake et al., patients with RFBs classified as colonic had more than double the likelihood of requiring operative intervention than those with objects confined to the rectum [13]. The anatomical anal canal, designated as the dentate line to the anal verge, measures an average of 2.1 cm in length in adult males and females [28]. The rectum, described as the rectosigmoid junction to the dentate line, measures approximately 12-15 cm in length [29]. Therefore, objects that are significantly greater than the combined anal canal and rectum length may have a higher likelihood of lodging into the colon. Similar findings have also shown an increased likelihood of surgical intervention for objects larger than 10 cm 13. Unfortunately, the specifics of the size of retrieved objects are not usually documented by the physician in the patient's chart. Another potential explanation could be the level of operative experience and environmental effects, including the level of sedation and comorbidities. One study noted an 89% failure rate of attempted bedside extractions, which led to the object traveling further up and requiring extraction in the OR [30]. These studies highlight the importance of assessing RFB location and ergonomics prior to retrieval. The use of ultrasound, X-rays, and computed tomography has been recommended to further explore the details of the inserted object and the possible presence of pathologies [11]. As such, we have noted that there are 20% of complications from mild to severe when operative interventions are required. 

Cologne et al. suggested that bedside transanal extraction of RFB should be attempted first, using local anesthesia with or without procedural sedation [6]. If not successful at the bedside, non-palpable RFBs should be evaluated and removed endoscopically in the OR, followed by laparotomy or laparoscopy [6]. Notably, a high percentage of RFBs in this study, with similar descriptions, were extracted digitally or by utilizing surgical tools in the OR under general anesthesia. The effect of general anesthesia on anal sphincter tone was associated with a greater decrease in anal pressure [31]. Furthermore, the success of digital or forceps retrieval methods reported in the OR prior to endoscopic or laparoscopic use may be due to the use of general anesthesia, including the use of muscle relaxants in relaxing the colonic and rectal tones, allowing the object to slide outward. From our study, we have noted a 40% success rate with the use of digital and forceps/anoscopy followed by 26% using sigmoidoscopy. The remainder of the studies required exploratory laparotomy and colectomy.

Interestingly, this study noted a disproportionately higher percentage of whites (39.7%) presenting to this hospital with RFBs. The racial distribution in San Bernardino County is as follows: 54.4% of residents identified as Hispanic or Latino, 27.3% White, 9.4% Black/African American, 8.0% Asian, 2.1% American Indian, and 0.5% Pacific Islander [32]. A prior study on racial demographics also noted a disproportionately higher percentage of Whites [33]. While no explanation was provided by the authors, one possible explanation for these unexpected results is that patients may be transiently seeking help from their region of residence to avoid a sense of humiliation [12,34]. An additional explanation is that, as a result of socioeconomic barriers to healthcare, patients may seek treatment elsewhere [34].

Like many other retrospective chart review studies, the current study has inherent limitations, which may include a limited patient history and methods of management. Electronic health records often lack details about the type, size, and tools used to remove RFB. In most cases, multiple attempts by various providers to remove RFB have been noted, but detailed information may not be recorded. In addition, some clinicians may not include detailed information or proper ICD codes, which may cause these cases to be excluded. However, efforts have been made to ensure an exhaustive search of electronic health records. Another limitation is the lack of long-term follow-up in most cases. This study was also limited by its small sample size. However, the majority of the present literature consists of studies with small sample sizes. Given that RFBs are infrequent, generating a large sample size is a significant challenge. Future prospective multicenter studies are needed to shed light on some of the limitations of this study.

## Conclusions

Management of rectal foreign bodies can be difficult based on the patient's presentation and the level of expertise available in the hospital. To improve the success rate of bedside retrieval and decrease complications, physicians need to be vigilant, communicative, and compassionate in their evaluations and clinical methodology.
